# An Account of the Æquilibrial Treatment Employed for the More Successful Union of Fractures of the Thigh-Bone, and the Prevention of Any Shortening of the Limb

**Published:** 1843-10

**Authors:** 


Art. V. —*Darstellung der Aequilibrial-Methode zur sicheren Heilung der Oberschenkelbrüche ohne Verkürzung.* Von Georg Mojsisovics, Doctor der Medicin und Chirurgie, Primarchirurgen im k. k. allgemeinen Krankenhause.—Wien, 1842. 8vo, pp. 114.

*An account of the Æquilibrial Treatment employed for the more successful union of Fractures of the Thigh-Bone, and the Prevention of any Shortening of the Limb.* By George Mojsisovics, Doctor of Medicine and Surgery, Senior Surgeon of the Imperial Hospital at Vienna, &c.—*Vienna*, 1842.

This essay is of a purely practical nature, and contains the experience of the writer on the relative advantages and faults of the various and common modes of treating fractures of the thigh-bone, with an examination of the anatomical reasons of the various forms of displacement from the direction of the fracture, the action of the muscles, and the weight of the limb. A short outline is also added of the influence of accidental causes, as varieties of health, alterations of temperature and concomitant diseases, in retarding the union of the broken limb.

This list of contents necessarily embraces a large field of inquiry, which might perhaps seem rather cramped for space in a pamphlet; but the short practical style and complete absence of all controversial writing, have enabled the author to compress his observations into a small compass.

The peculiarity of the author's views consists in the peculiar apparatus which he has introduced and found exceedingly successful in the treatment of broken thighs. The other part of the work, though valuable for its sound sense and practical value, does not contain *novelties*, but rather a condensed view of the present *knowledge* of this subject, corrected by the writer's experience. To the novel part of this pamphlet we therefore now proceed:

The long splint of Desault and Mr. Liston, the double inclined planes of Sir Charles Bell and others, or the fracture bed of Mr. Earle, are all used in this country for fractures of the thigh. In the great mass of cases union is effected in a complete manner, and the patient recovers with a useful and well-shaped limb. When the fracture occurs below the upper third of the bone, the success is however greater than when the fracture is higher up. And it certainly must be confessed that the broken thigh does not always exactly assume its old form after union from fracture.

The limb may be short, or the lower portion inside the upper in fractures of the upper third of the thigh, the lower extremity of the upper portion is especially liable to jut forwards, whilst the upper extremity of the lower portion passes behind it; the lower portion is also not unfrequently rotated unduly outwards. Such, in short, are the most important faults after fracture of the thigh, producing shortening of the limb, more or less eversion of the foot, as well as alteration on the lateral and antero-posterior axes of the limb.

Whether the fracture be oblique or transverse, Dr. Mojsisovics' object is to obtain such accurate union that the femur shall retain the exact form of health. The form and situation of the fracture, also the number of fractures, if more than one, exercise a certain influence on the position of the part, but the muscles passing from the pelvis to the lower extremity form the chief obstacle to a proper adjustment of the broken parts, and have chiefly attracted the notice of our author.

The muscles passing from the pelvis, as well as from the loins to the thigh-bone and knee, form, according to the writer, a cone, the base of which is above and the apex below. These muscles are but little under the control of bandages, and are liable to draw the lower fragment away from the upper, as well as to bend the thigh-bone upon the pelvis. Such, in short, are the general results of the action of the muscles which form the obstacle to accurate union, and which impediment the writer removes by his new apparatus. We need not give a minute detail of this, as its nature will be well enough understood by a general description.

The patient is placed on a firm bed, over which a framework is laid; this frame consisting of four posts connected by a horizontal framework. In short, let the reader picture to himself a common four-post bed, the posts and connecting transverse upper beams of which are very strong.

The patient is laid flat on his back, whilst a strong band, passed from one side of the bed to the other, fixes his pelvis on the bed firmly. The thighs are then bent at right angles to the body and placed in a vertical position, whilst the legs are bent at the knee, and consequently placed in a horizontal position at right angles to the thighs. Two broad folds of linen, passed from the upper beams of the frame beneath the calves of the legs, support them ; pasteboard splints, fitting accurately to the thighs and buttocks, keep them in position; whilst a weight, passed over a pulley and fastened to the foot of the broken limb, extends the limb to the level of the sound limb placed by its side. The patient, in short, lies on his back in bed with his legs in the same relation to his body that they are when he is sitting in a chair, but now necessarily bent up in the air.

This position at first seems strange, but, on reflection, it will be found to deserve most serious attention : we think the plan reflects credit on the inventor.

Under the care of Dr. Mojsisovics all fractures of the thigh are thus successfully treated. Such a general use of this plan cannot be expected in this country, but in the treatment of fractures of the upper third of the thigh it deserves careful trial. Great difficulty is often experienced in bringing down the prominent end of the upper portion, but this plan of bringing up the lower portion to the upper certainly appears to remove the difficulty, and is likely to place the upper and lower portions in the same line, whilst their overlapping is prevented by the extension at the foot.

This mode of treatment presents nothing to render it dangerous or questionable, but unites a means of accurate extension with complete rest and support to the broken limb; superior, according to the writer's experience, to the common modes of treatment; and certainly, to all appearance, equally successful with them in the majority of cases of fracture of the thigh, and superior to many in the treatment of fractures of the upper third of that bone.

